# Supplementary material for: The molecular basis of regulation of bacterial capsule assembly by Wzc
Source: Nat Commun. 2021 Jul 16;12:4349. doi: 10.1038/s41467-021-24652-1 (PMC8285477; doi:10.1038/s41467-021-24652-1)

The molecular basis of regulation of bacterial capsule assembly by Wzc

Yun Yang[1,2,3#], Jiwei Liu[1],[2#], Bradley R Clarke[4], Laura Seidel[4], Jani R. Bolla[5,6], Philip N. Ward[1,2,3], Peijun Zhang[2,7], Carol V. Robinson[5,6], Chris Whitfield[4]* and James H Naismith[1,2,3]*

1 Rosalind Franklin Institute, Harwell Campus, UK OX11 0FA

2 Division of Structural Biology, Roosevelt Drive, The University of Oxford, OX3 7BN

3 The Research Complex at Harwell, Harwell Campus, UK OX11 0FA

4 Department of Molecular and Cellular Biology, The University of Guelph, Ontario, Canada N1G 2W1

5 Physical and Theoretical Chemistry Laboratory, Department of Chemistry, South Parks Road, The University of Oxford, OX1 3QZ

6 The Kavli Institute for Nanoscience Discovery, South Parks Road, Oxford, OX1 3QU

7 Electron Bio-Imaging Centre, Diamond Light Source, Harwell Science and Innovation Campus, UK OX11 0DE

[#] Equal contribution

* Email for correspondence to Chris Whitfield cwhitfie@uoguelph.ca or James Naismith naismith@strubi.ox.ac.uk

Supplementary Table 1. Cryo-EM data collection, refinement and validation statistics.

	Wzc^{K540M} Apo		Wzc^{K540M} peri	Wzc^{K540M} ADP		Wzc^{K540M}4YE Apo	
	EMDB-12338<br>PDB-7NHR	EMDB-12339<br>PDB-7NHS	EMDB-12340	EMDB-12360<br>PDB-7NII	EMDB-12359<br>PDB-7NIH	EMDB-12353<br>PDB-7NIB	EMDB-12349<br>PDB-7NI2
**Data collection and processing**							
Magnification	105,000	105,000	105,000	105,000	105,000	105,000	105,000
Voltage (kV)	300	300	300	300	300	300	300
Electron exposure (e–/Å^2)	55	55	55	53.6	53.6	57.5	57.5
Defocus range (μm)	-0.5 ~ -2.5	-0.5 ~ -2.5	-0.5 ~ -2.5	-0.5 ~ -2.5	-0.5 ~ -2.5	-0.5 ~ -3.0	-0.5 ~ -3.0
Pixel size (Å)	0.829	0.829	0.829	0.829	0.829	0.831	0.831
Symmetry imposed	C1	C8	C1	C1	C8	C1	C8
Initial particle images (no.)	5,162,225	5,162,225	5,162,225	1,268,401	1,268,401	1,735,033	1,735,033
Final particle images (no.)	148,431	546,088	148,431	261,748	261,748	71,319	71,319
Map resolution (Å)	2.85	2.30	2.77	2.88	2.60	3.51	2.89
FSC threshold	0.143	0.143	0.143	0.143	0.143	0.143	0.143
Map sharpening *B* factor (Å^2)	-84.56	-94.65	-72.34	-94.76	-110.96	-91.69	-113.99
**Refinement**							
Model resolution (Å)	2.9	2.4		3.0	2.6	3.6	3.0
FSC threshold	0.5	0.5		0.5	0.5	0.5	0.5
Model composition							
Non-hydrogen atoms	37011	32240		37235	32464	35037	31344
Protein residues	4772	4176		4772	4176	4543	4080
Ligands				16 (8 ADP, 8 Mg^{2+})	16(8 ADP, 8 Mg^{2+})		
B factors (Å^2)							
Protein	57	30		39	27	114	75
Ligand				18	14		
R.m.s.d.							
Bond lengths (Å)	0.006	0.002		0.008	0.010	0.007	0.007
Bond angles (°)	0.691	0.468		0.66	0.699	0.775	0.687
Validation							
MolProbity score	1.73	1.50		1.82	1.68	1.95	1.77
Clashscore	9.22	5.78		12.20	7.52	14.59	11.52
Poor rotamers (%)	0.00	0.00		0.00	0.00	0.00	0.00
Ramachandran plot							
Favored (%)	96.4	96.9		96.6	96.1	96.0	96.8
Allowed (%)	3.6	3.1		3.4	3.9	4.0	3.2
Disallowed (%)	0.0	0.0		0.1	0.0	0.0	0.0

Supplementary Figure 1. ***In vitro*** **analysis of wild-type Wzc and Wzc^{K540M}.**
a, SDS-PAGE of purified full-length wild-type (WT) Wzc. Experiments were performed in biological triplicate and were consistent, a representative experiment is shown.
b, Gel filtration of purified WT Wzc.
c, Negative staining micrograph of WT Wzc. A red square outlines zoom inset in the upper right corner. The scale bars in the full micrograph and zoom inset are 200 nm and 30 nm respectively, same for f and j. Multiple micrographs were prepared and examined, an example is shown.
d, Proteins were western blotted with anti-phosphotyrosine (anti-pTyr) and anti-polyHistidine (anti-His) antibodies. Wzb treated Wzc was incubated at room temperature for 0, 1, 2 and 3 hours (hr). The uncropped images are available in Supplementary Figure 10c. Experiments were performed in biological triplicate and were consistent, a representative experiment is shown.
e, Gel filtration of purified WT Wzc treated with Wzb at room temperature for 3 hours.
f, Negative staining micrograph of Wzb treated Wzc. Multiple micrographs were prepared and examined, an example is shown.
g, , Left panel: Cryo-EM micrograph of Wzb treated Wzc showing the presence of octamer, which represents a small portion. The green circles show the auto-picking positions. The representative octamer is labelled with red arrow and zoomed next to it. The scale bars in the full micrograph and zoomed inset are 50 nm and 15 nm respectively. Multiple micrographs were prepared and examined, an example is shown. Right panel: 2D class averages of octamers. Box size, 248 Å.
h, SDS-PAGE of purified Wzc^{K540M}. Experiments were performed in biological triplicate and were consistent, a representative experiment is shown.
i, Gel filtration of purified Wzc^{K540M}.
j, Negative staining micrograph of purified Wzc^{K540M}. Multiple micrographs were prepared and examined, an example is shown.

Supplementary Figure 2. Mass spectrometry identifies the phosphorylation of the C-terminal tyrosine-rich tail.
a. Phosphoproteomics analysis identify the phosphorylation sites. A list of phosphopeptides with a score of more than 100 are shown here and phospho site probabilities are shown in the parenthesis. The species with the four conserved phosphorylated tyrosines (Y708, Y713, Y715, Y717) is the most abundant. The pattern suggests the enzyme does not have to always start at Y717 and can skip intervening residues. We do not know if there is an obligate order to phosphorylation.
b. MS/MS spectra of the phosphopeptide YGHNHYGp**Y**Sp**Y**p**Y**DKK show rich fragmentation in which phosphorylation sites on Y715, Y717 and Y718 were detected. Peaks in orange correspond to the loss of water or ammonia.
c. Native mass spectrometry analyses indicate that the tyrosine mutants are monomeric and are differently phosphorylated. Only charge state 9+ is shown here for clarity. The spectra show how the number of phosphorylations changes alongside the number of Y to E mutations, which is further supported by proteomics analyses. Number of phosphosites identified within the peptide 708-718 are shown on the right.

Supplementary Figure 3. Cryo-EM analysis of apo Wzc^{K540M}.
a**,** Overview of cryo-EM data collection and image processing workflow for apo Wzc^{K540M}.
b, Representative micrograph of apo Wzc^{K540M} recorded by K3 detector. Some representative particles are labelled with green circles. Scale bar = 50 nm. Multiple micrographs were prepared and examined, an example is shown.
c, Representative 2D class averages of Wzc^{K540M} octamer. Box size = 312 Å.
d, Local resolution estimation of apo Wzc^{K540M} with C1 symmetry.
e, FSC curves of apo Wzc^{K540M} with C1 symmetry, generated by cryoSPARC.
f, Local resolution estimation of apo Wzc^{K540M} with C8 symmetry.
g, FSC curves of apo Wzc^{K540M} with C8 symmetry, generated by cryoSPARC.
h, Local resolution estimation of Wzc^{K540M} periplasmic domain after local refinement.
i, FSC curves of Wzc^{K540M} periplasmic domain after local refinement, generated by cryoSPARC**.**
j-m, Cryo-EM densities for representative regions: cytoplasmic domain (CD) (**j**), periplasmic domain (PD) motif 1 (**k**) and motif 2 (**l**), transmembrane helices (**m**).

Supplementary Figure 4. ADP-bound Wzc^{K540M} local resolution maps and FSC curves.
a, Local resolution maps of ADP-bound Wzc^{K540M} with C1 symmetry.
b, FSC curves of ADP-bound Wzc^{K540M} with C1 symmetry, generated by cryoSPARC.
c, Local resolution maps of ADP-bound Wzc^{K540M} with C8 symmetry.
d, FSC curves of ADP-bound Wzc^{K540M} with C8 symmetry, generated by cryoSPARC.
e, Cryo-EM density (grey) of bound ADP and Mg^{2+} at the active site. ADP is shown in sticks coloured by atoms and the associated Mg^{2+} as a magenta sphere. Surrounding residues are labelled and shown as sticks.

Supplementary Figure 5. Sequence alignment among bacterial tyrosine kinases.
a, Sequence alignment of bacterial tyrosine kinase from 16 strains, in which 13 are Gram-negative bacteria and the last 3 are Gram-positive bacteria. Wzc_K30 is the protein used in this study and its secondary structure is shown on top of the aligned sequences. Active-site residues K540, T541, D562, D642, P645 and N696 are either conserved or conservatively substituted in both Gram-negative and Gram-positive PCP proteins, while P644 is not absolutely conserved in Gram-positive representatives. Residues S475, F542 and Y569 are conserved among Gram-negative examples.
b, Sequence alignment of Gram-positive tyrosine kinase membrane adaptor (last 6 sequences), which is homologous to the Gram-negative tyrosine kinase N-terminal region (including periplasmic and transmembrane regions, roughly 1-450 in Wzc_K30). Periplasmic motif 2 and 3 are missing in Gram-positive bacteria. The alignment results are prepared with ESPript 3.0 server (http://espript.ibcp.fr/ESPript/ESPript/).

Supplementary Figure 6. Structural comparison of Wzc and Wzz.
a, Wzc has three periplasmic motifs. Motif 1 is structurally conserved in Wzz. Motif 2 is absent from Wzz and motif 3 (shown here in class i conformation) has a completely different structure. Secondary structure elements are labelled. Colors are as Figure 2a.
b, The octamer of full-length Wzc and Wzz PDB 6RBG[1] are very different outside of the membrane.
c, Seen from the periplasm the octamer of Wzc is larger than Wzz[1].

Supplementary Figure 7. Cryo-EM structure of periplasmic motif 3.
a, 3D classification and 3D refinement generate at least three groups in terms of the class i motif 3 visible in the map.
b, Side view (panel above) and top view (panel below) of the cryo-EM map densities of periplasmic motif 3.
c-e, Representative map densities for motif 3 of chain A (**c**), chain B (**d**) and chain C (**e**).
f, Superposition of chain B, C, G and H show the offset of helices among each other, in which chain B, G are similar, and chain C, H are similar. Chain B, G are different from chain C, H.

Supplementary Figure 8. ***In vitro*** **and** ***in vivo*** **functional analysis of C-terminal tyrosine rich tail.**

a, Gallery of negative-stain micrographs of Wzc^{K540M} with different Y(Tyr) to E (Glu) mutants showing the oligomerization states. Red square outlines zoom inset on the corner. Scale bars in the whole micrograph and zoomed inset are 200 nm and 30 nm respectively. The representative octamers in micrographs are labelled with red circles. A rough ratio of octamers in each mutant is shown below the micrograph. The ratio is defined as the percentage of octamers inside total particles in the micrographs. The octamers are judged by the size and shape of the particles. Multiple micrographs were prepared and examined, an example is shown.

b, Western immunoblot of whole-cell lysates probed with anti-K30 antiserum (panel above, for detecting cell surface polysaccharides) and anti-His antibody (panel below, for detecting the expression of Wzc derivatives) showing the effect of single or double Y to E substitutions on the production of polysaccharide. Experiments were performed in biological triplicate and were consistent, a representative experiment is shown.

c, Detection of pTyr in purified Wzc proteins with anti-pTyr antibody. The loading amount of each lane was checked through anti-His antibody. Uncropped gels in Supplementary Figure 10d. Experiments were performed in technical triplicate and were consistent, a representative experiment is shown.

d, Western immunoblot of whole-cell lysates probed with anti-K30 antiserum (top panel), anti-His antibody (middle panel) and anti-pTyr antibody (bottom panel, for detection of pTyr) showing the effect of 2^{alt}YE (Y715E/Y717E), 3^{alt}YE (Y713E/ Y715E/Y717E) and 4^{alt}YE (Y708E/ Y713E/ Y715E/Y717E) mutants on the production of polysaccharide. Experiments were performed in biological triplicate and were consistent, a representative experiment is shown.

e, Detection of pTyr of purified wild type, 2^{alt}YE, 3^{alt}YE and 4^{alt}YE Wzc derivates. Uncropped gels in Supplementary Figure 10e. Experiments were performed in technical triplicate and were consistent, a representative experiment is shown.

f, The uncropped images as shown in Figure **4a**. Western immunoblot of whole-cell lysates probed with anti-K30 antiserum (top panel), anti-His antibody (middle panel) and anti-pTyr antibody (bottom panel) showing the effect of 3YE (Y715E/Y717E/Y718E), 4YE (Y713E/Y715E/Y717E/Y718E), 5YE (Y708E/Y713E/Y715E/Y717E/Y718E) and 7YE (Y705E/Y706E/Y708E/Y713E/Y715E/Y717E/Y718E) mutants on the production of polysaccharide. The band observed close to 25 kDa in the anti-His western blot corresponds to a cross-reactive protein present in *E. coli* K30. Experiments were performed in biological triplicate and were consistent, a representative experiment is shown.

Supplementary Figure 9. Cryo-EM structure of Wzc^{K540M}4YE and map quality validation.
a, SDS-PAGE gel of purified Wzc^{K540M}4YE. Experiments were performed in biological triplicate and were consistent, a representative experiment is shown.
b, Representative micrograph of Wzc^{K540M}4YE recorded by K3 detector. Some representative particles are labelled with green circles. Scale bar = 50nm. Multiple micrographs were prepared and examined, an example is shown.
c, Representative 2D class averages of Wzc^{K540M}4YE octamer. Box size = 312 Å.
d, Representative 2D class averages of dissociated particles from Wzc^{K540M}4YE sample. Box size = 312 Å.
e, Bottom (left), side (middle) and top (right) views of cryo-EM structure of Wzc^{K540M}4YE at C1 symmetry.
f, Local resolution estimation of Wzc^{K540M}4YE in C1 symmetry.
g, FSC curves of Wzc^{K540M}4YE in C1 symmetry, generated by cryoSPARC.
h, Local resolution estimation of Wzc^{K540M}4YE in C8 symmetry.
i, FSC curves of Wzc^{K540M}4YE in C8 symmetry, generated by cryoSPARC.
j, Cryo-EM densities for the C-terminal tail of Wzc^{K540M}4YE.

Supplementary Figure 10. Uncropped gels that are shown in this study, red denotes cropped region.
a, Blots shown in Figure 4b.
b, Blots shown in Figure 5b.
c, Blots shown in Supplementary Figure 1d.
d, Blots shown in Supplementary Figure 8c.
e, Blots shown in Supplementary Figure 8e.

References

1. Wiseman, B., Nitharwal, R. G., Widmalm, G. & Högbom, M. Structure of a full-length bacterial polysaccharide co-polymerase. *Nat Commun* **12**, 369 (2021).

Supplementary Figure 1

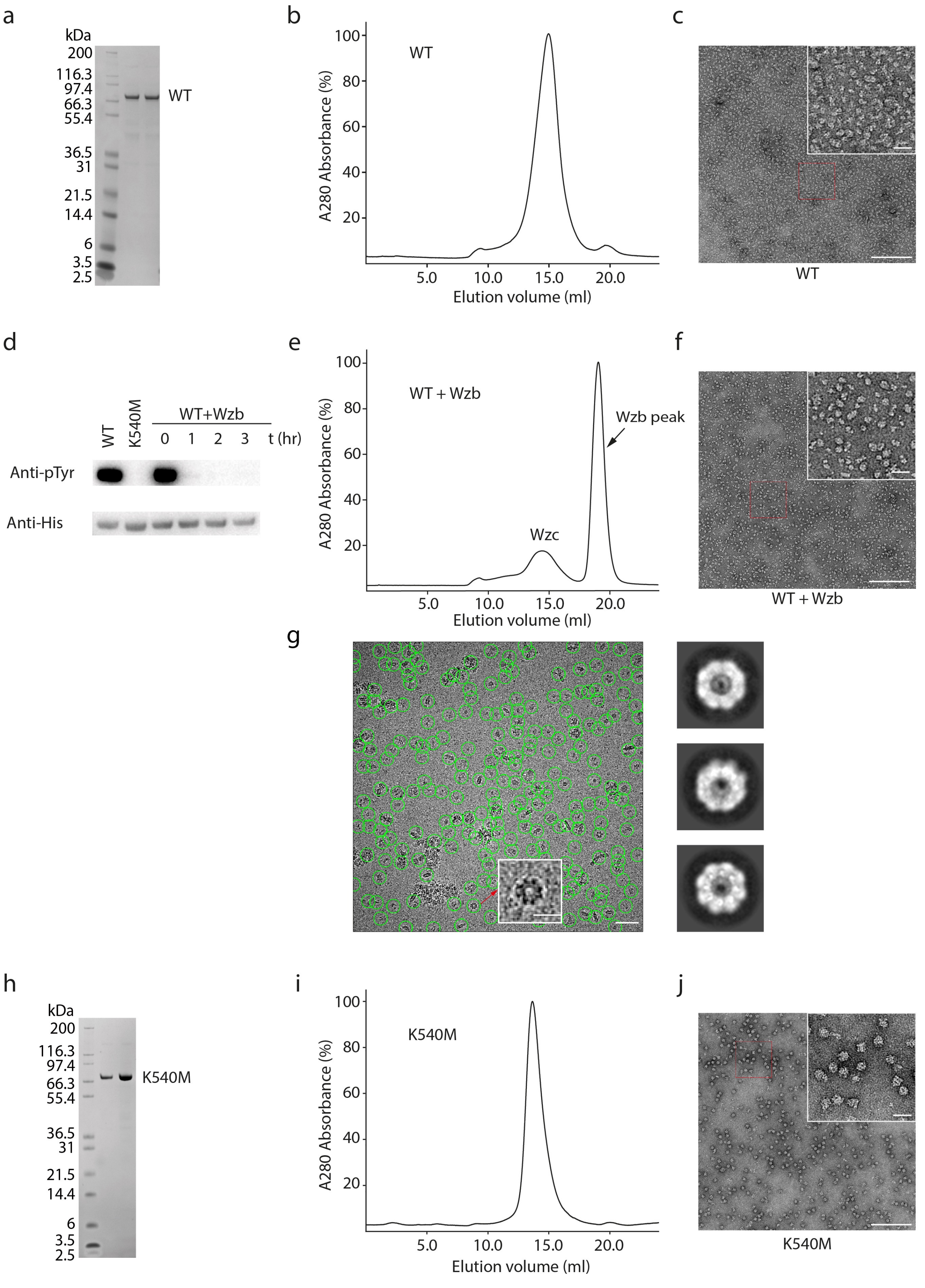

Supplementary Figure 2

a

Peptide sequence (phospho score probabilities)	Phospho site position	PEP score	Score	*z*	Mass error (ppm)	*m/z*
p**Y**(1)GHNHY(0.248)Gp**Y**(0.752)Sp**Y**(0.933)Y(0.067)DKK	Y708,<br>Y715,<br>Y717	6.28E-91	233.69	2	0.4855	1017.85
p**Y**(1)GHNHp**Y**(1)Gp**Y**(0.995)Sp**Y**(0.795)Y(0.21)DKK	Y708,<br>Y713,<br>Y715,<br>Y717	6.93E-60	200.63	3	-0.0485	705.56
ISHILDSISQNp**Y**(1)LAQNIAR	Y255	1.96E-54	185.37	2	0.28172	1118.56
YGHNHY(0.251)Gp**Y**(0.748)Sp**Y**(1)p**Y**(1)DKK	Y715,<br>Y717,<br>Y718	5.34E-38	178.07	3	0.1569	678.90
p**Y**(1)GHNHY(0.005)Gp**Y**(0.994)SY(0.065)p**Y**(0.935)DKK	Y708,<br>Y715,<br>Y718	3.85E-38	175.49	2	-0.8371	1017.85
p**Y**(1)GHNHp**Y**(0.994)GY(0.006)Sp**Y**(1)p**Y**(1)DKK	Y708,<br>Y713,<br>Y717,<br>Y718	6.88E-38	173.44	3	0.1395	705.56
ISHILDSISQNp**Y**(1)LAQNIAR	Y255	8.03E-53	173.03	3	0.3257	746.02
p**Y**(1)GHNHp**Y**(1)Gp**Y**(1)Sp**Y**(0.972)Y(0.028)DKK	Y708,<br>Y713,<br>Y715,<br>Y717	2.15E-37	165.78	2	-0.1392	1057.83
Y(0.076)GHNHp**Y**(0.908)GY(0.016)SY(0.138)p**Y**(0.862)DKK	Y713,<br>Y718	3.06E-29	158.08	2	-0.5261	977.86
Y(0.085)GHNHp**Y**(0.907)Gp**Y**(0.987)SY(0.222)p**Y**(0.799)DKK	Y713,<br>Y715,<br>Y718	6.58E-22	137.01	2	0.3879	1017.85
p**Y**(0.996)GHNHp**Y**(0.502)GY(0.502)Sp**Y**(0.955)Y(0.045)DKK	Y708,<br>Y713,<br>Y717	1.04E-13	109.11	2	-0.4808	1017.85
p**Y**(0.547)GHNHY(0.447)GY(0.006)SYYDKK	Y708	6.38E-13	108.43	2	-0.0416	873.83
p**Y**(1)GHNHp**Y**(1)Gp**Y**(1)Sp**Y**(1)p**Y**(1)DKK	Y708,<br>Y713,<br>Y715,<br>Y717,<br>Y718	2.02E-13	103.08	2	0.4209	1097.81

b

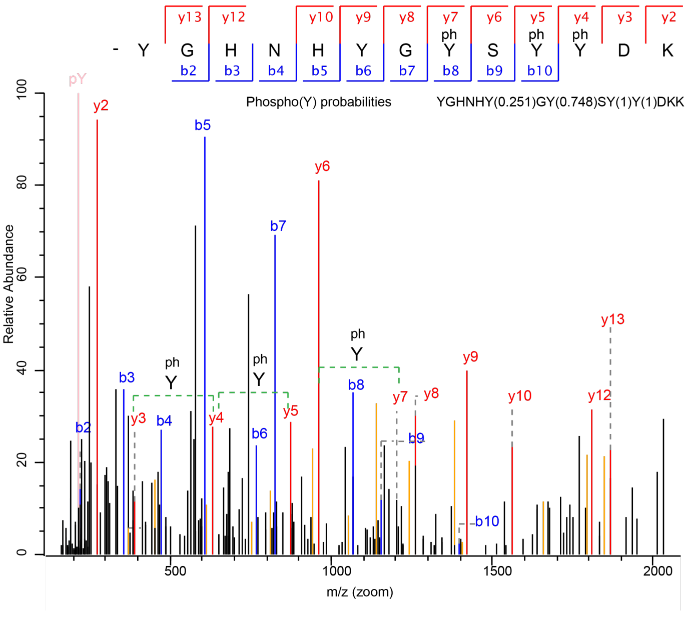

c

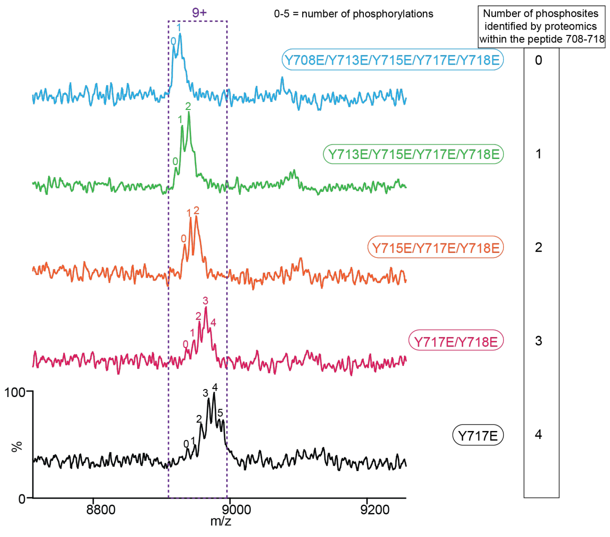

Supplementary Figure 3

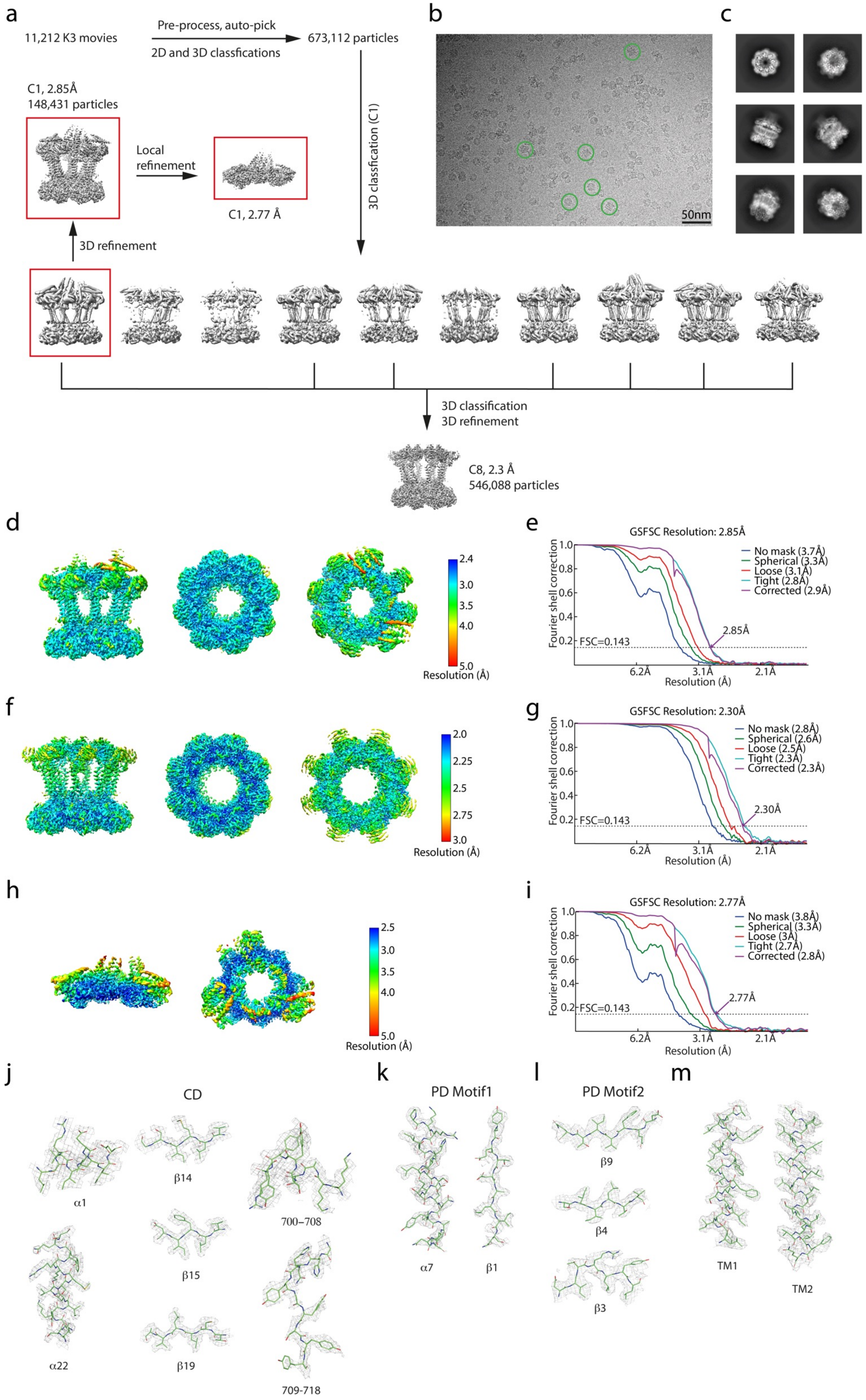

Supplementary Figure 4

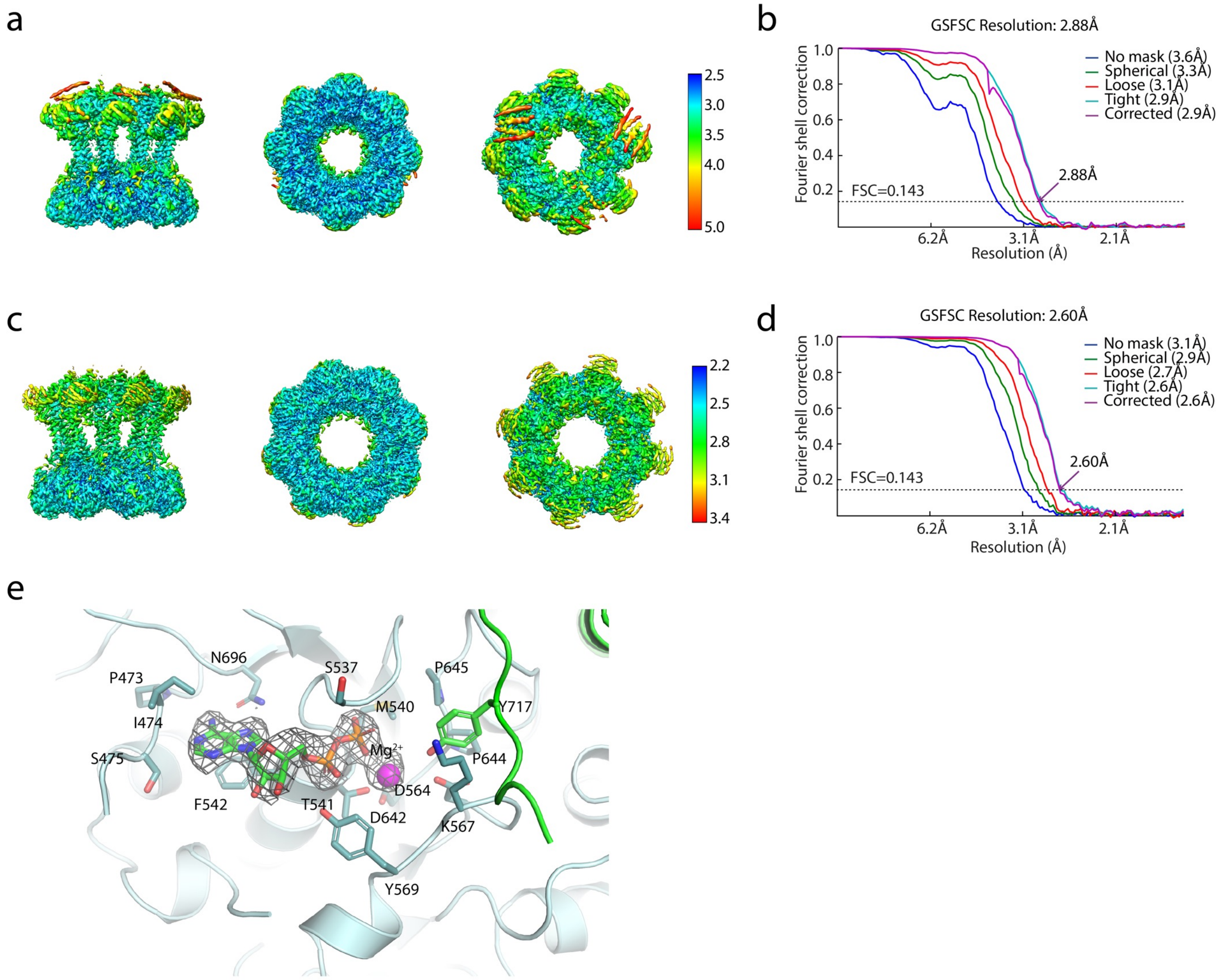

Supplementary Figure 5

a

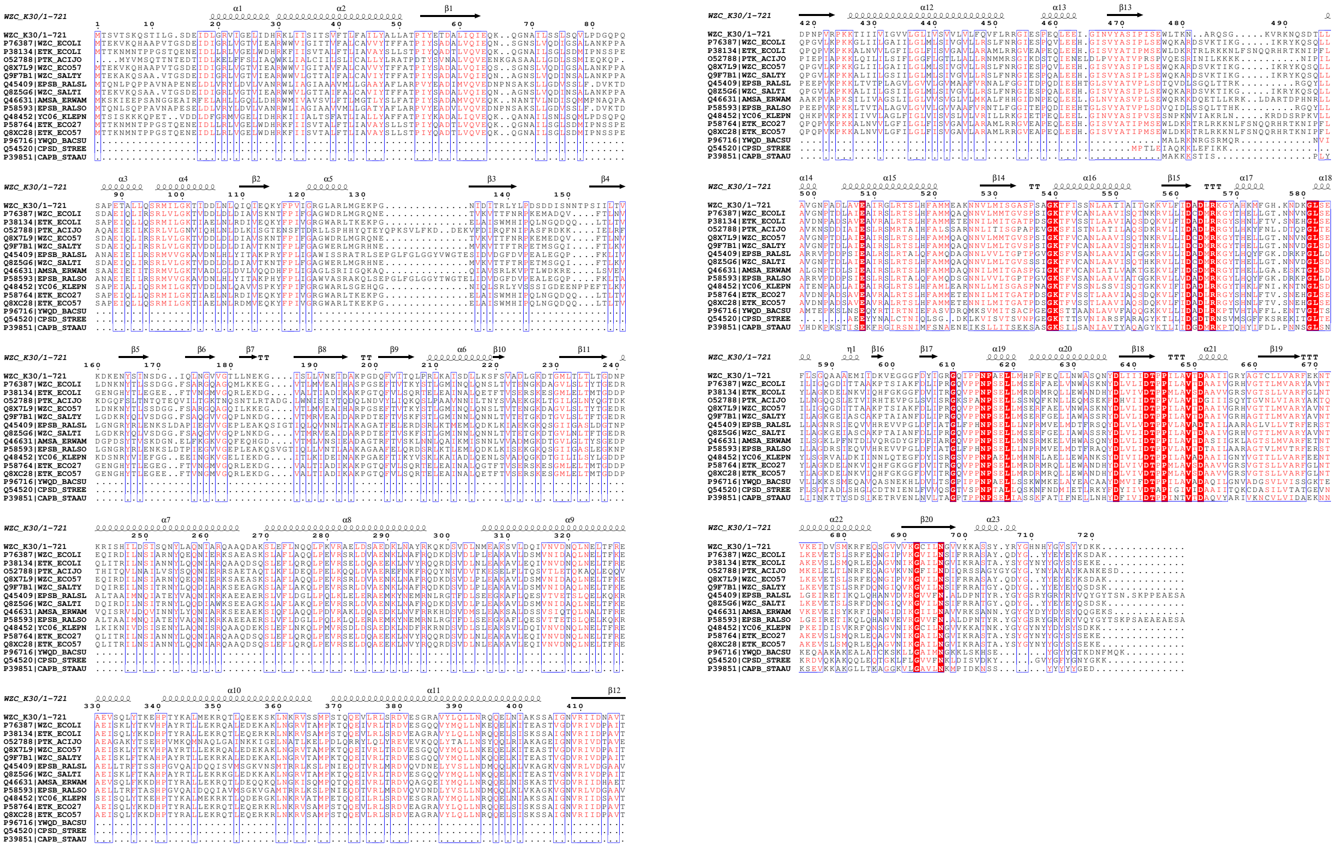

b

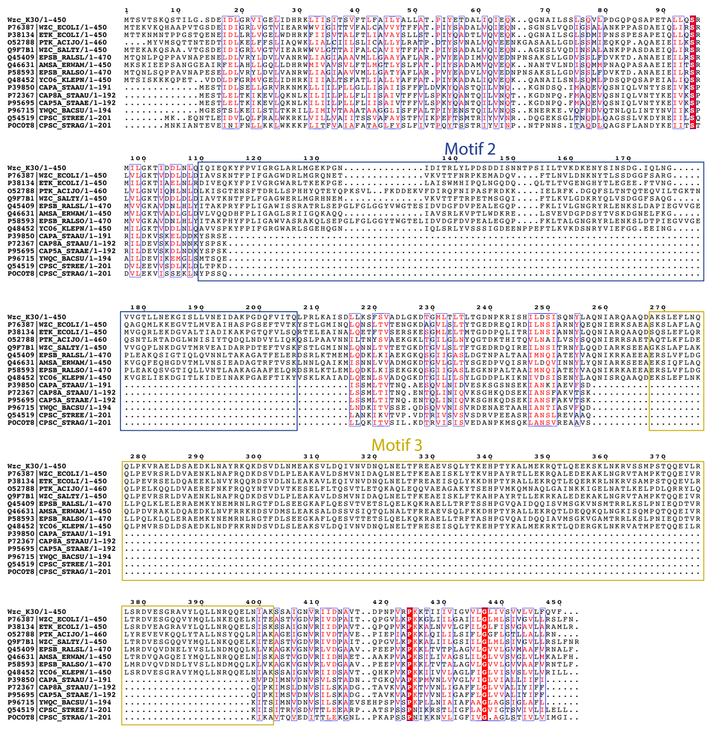

Supplementary Figure 6

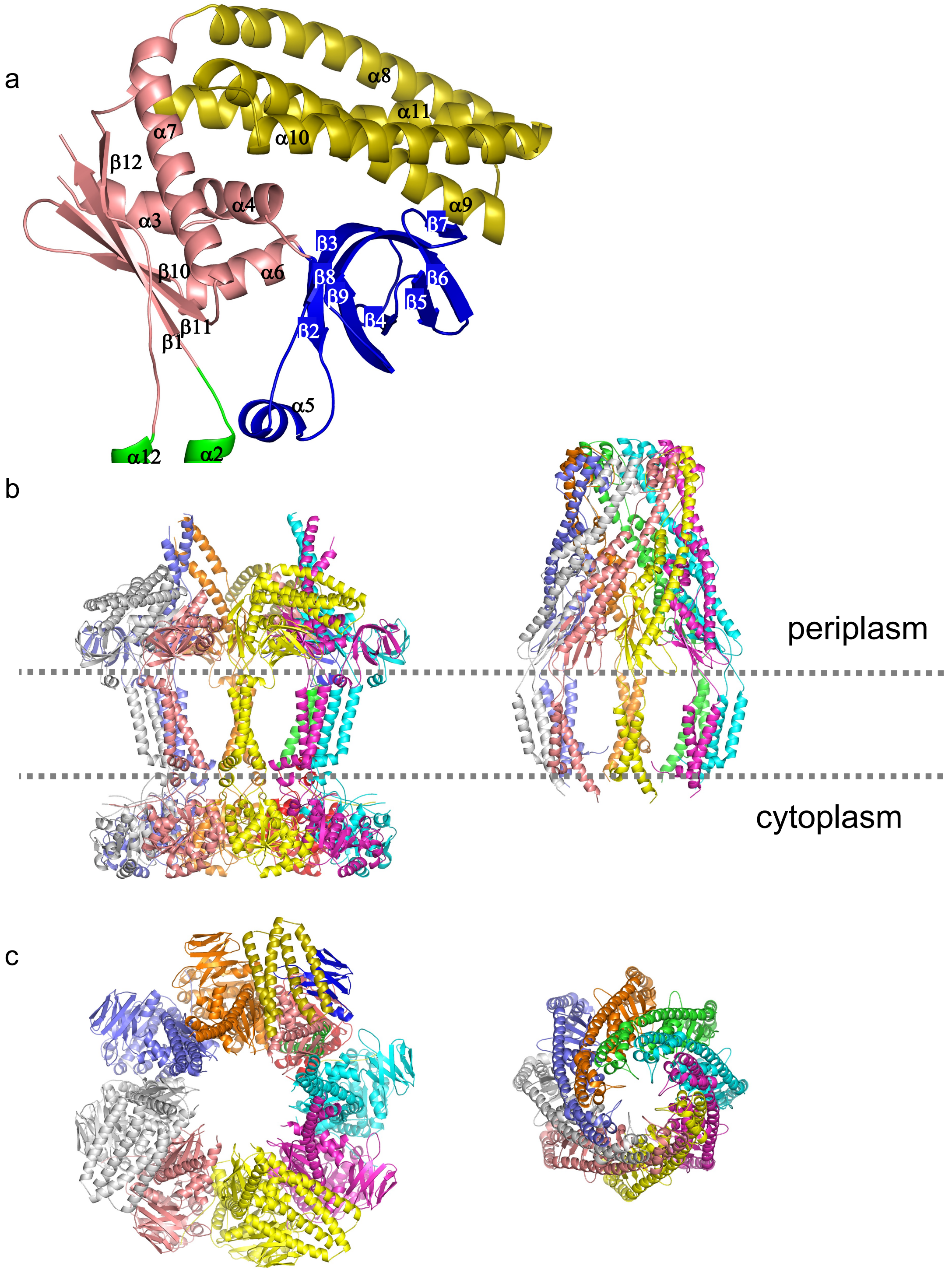

Supplementary Figure 7

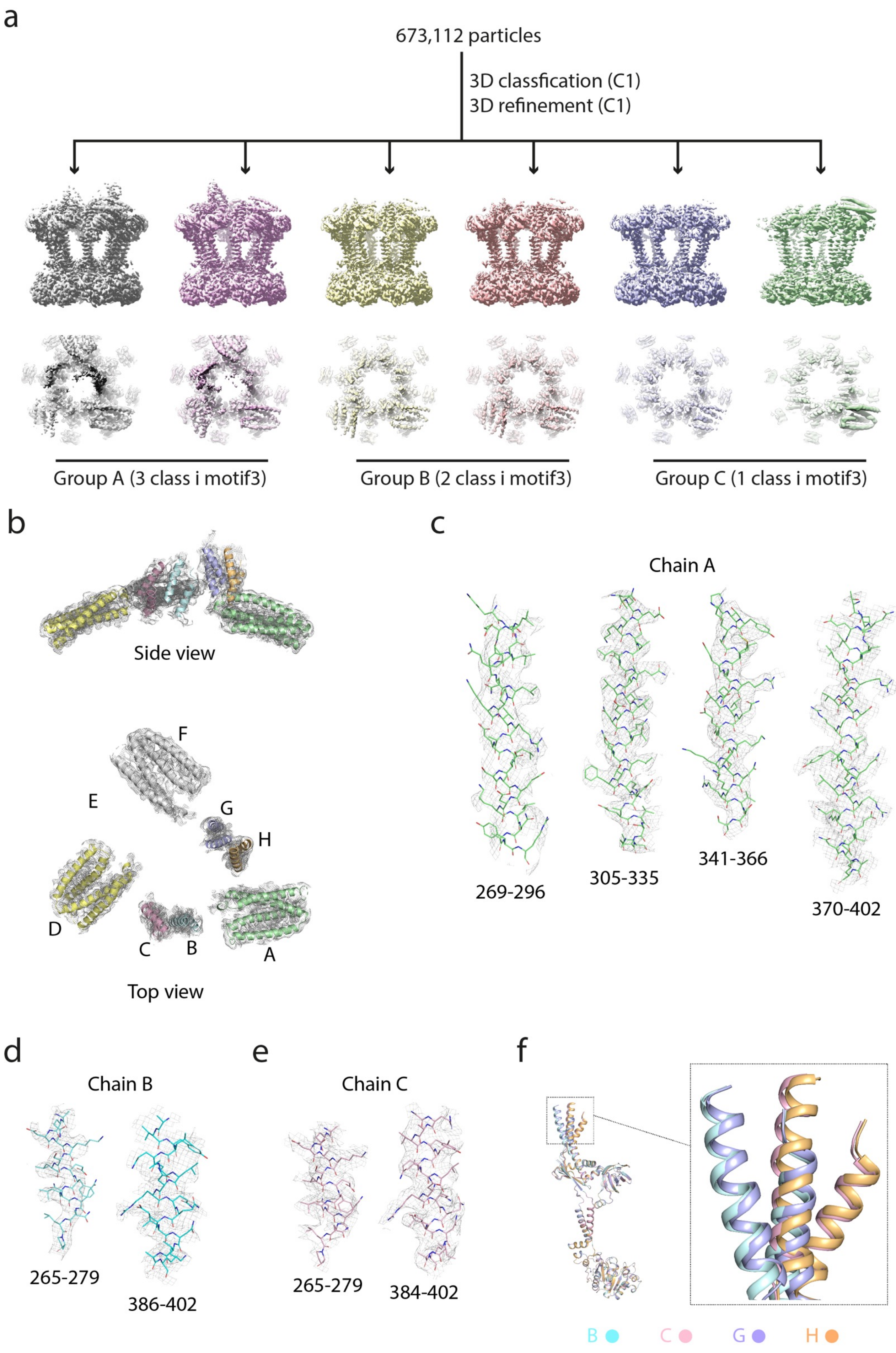

Supplementary Figure 8

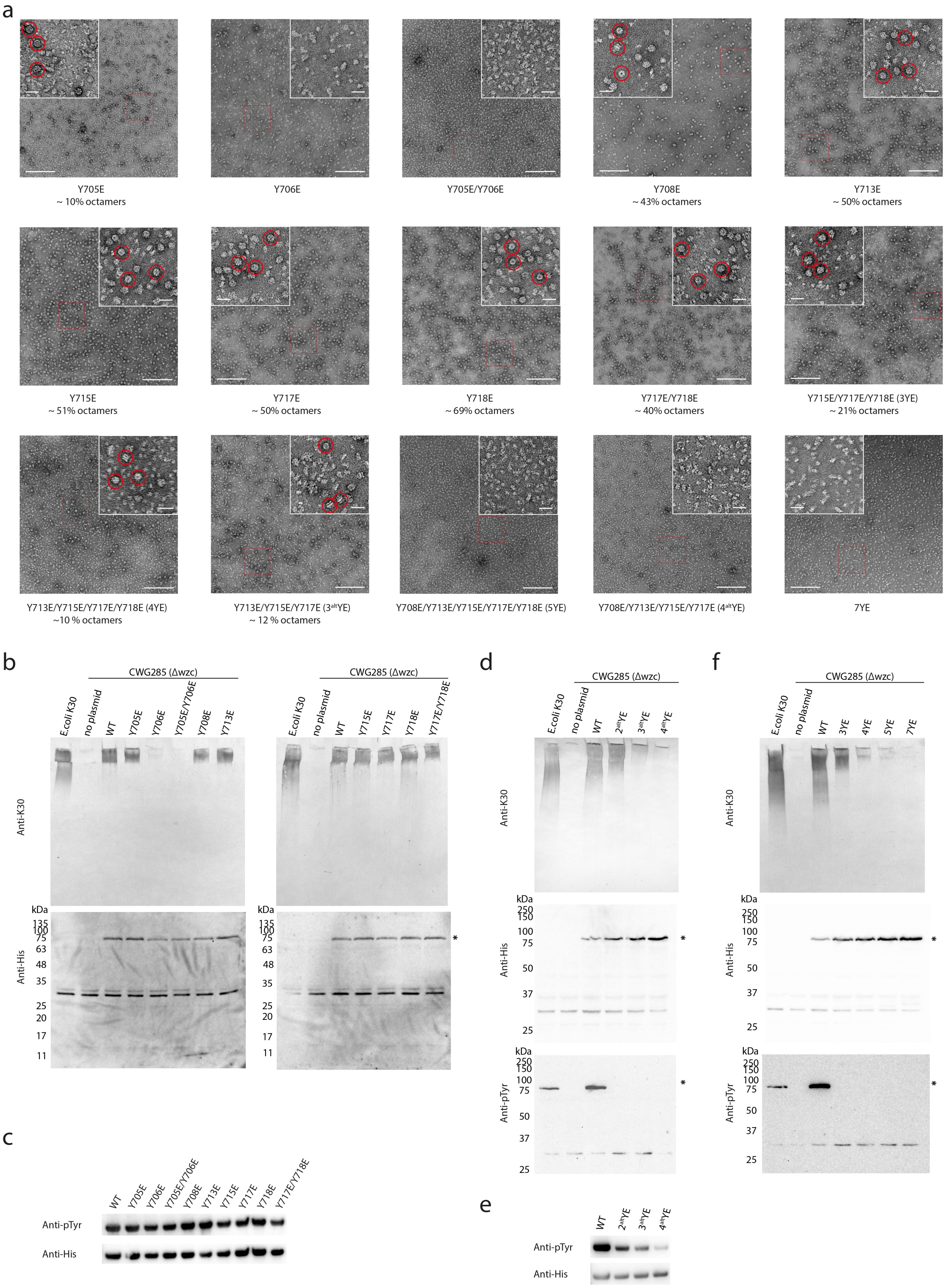

Supplementary Figure 9

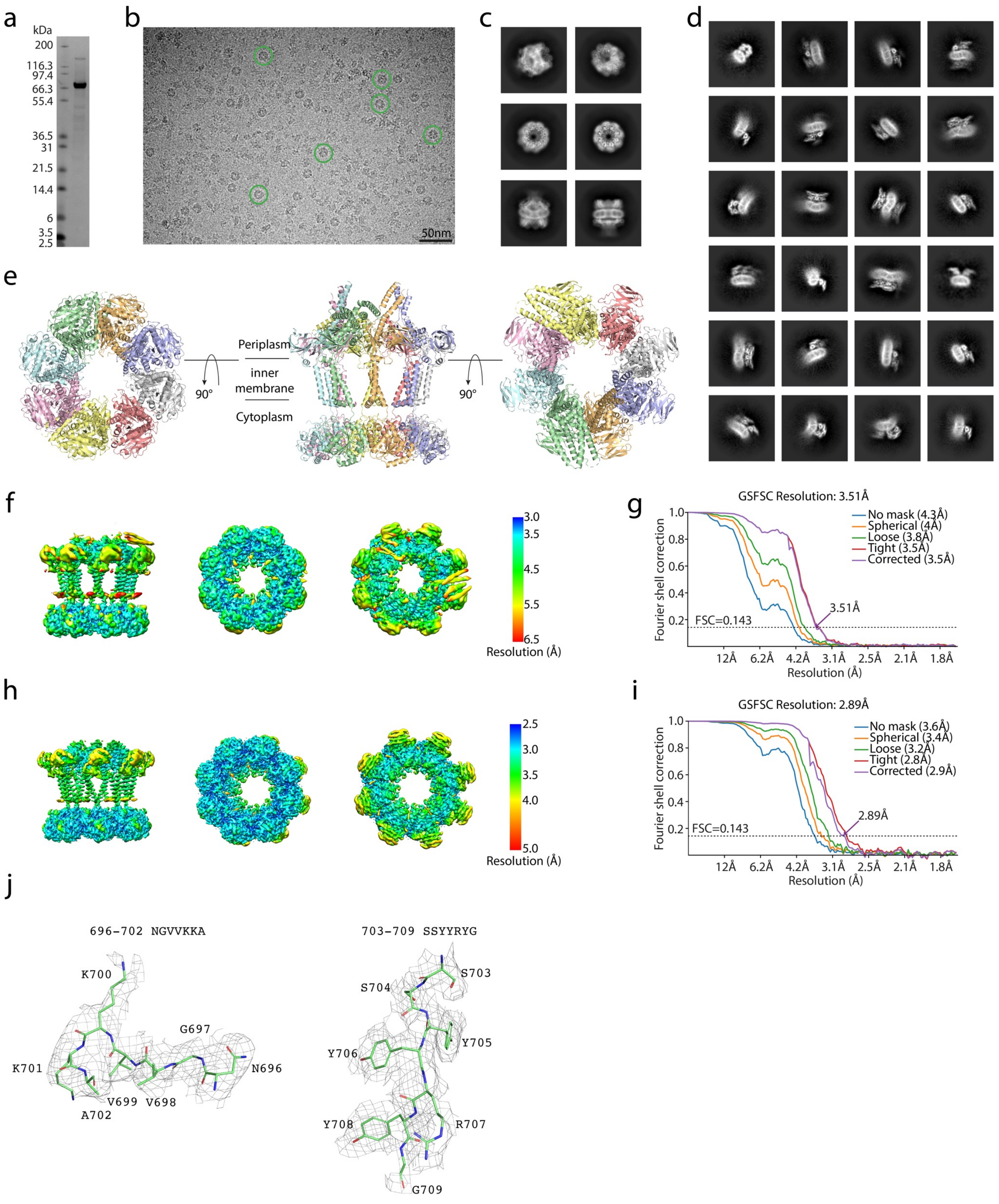

Supplementary Figure 10

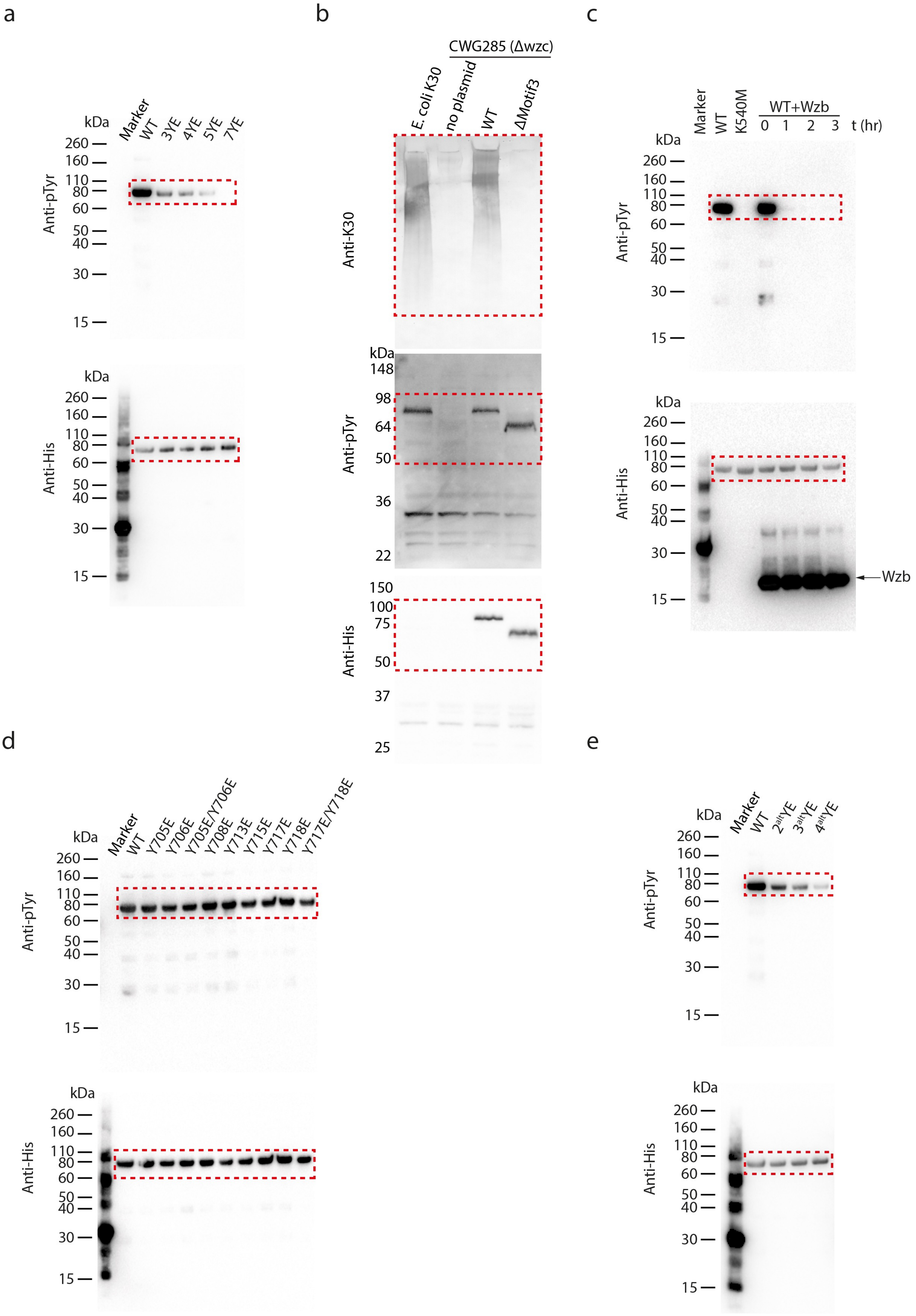

Supplement: Supplementary file 1 — Supplementary Information [file 41467_2021_24652_MOESM1_ESM.pdf]
