# Supplementary material for: The molecular basis of regulation of bacterial capsule assembly by Wzc
Source: Nat Commun. 2021 Jul 16;12:4349. doi: 10.1038/s41467-021-24652-1 (PMC8285477; doi:10.1038/s41467-021-24652-1)
Supplement: Supplementary file 3 — Reporting Summary [file 41467_2021_24652_MOESM3_ESM.pdf]

nature research

Corresponding author(s): Chris Whitfield & James H Naismith

Last updated by author(s): 24-June-2021

Reporting Summary

Nature Research wishes to improve the reproducibility of the work that we publish. This form provides structure for consistency and transparency in reporting. For further information on Nature Research policies, see our Editorial Policies and the Editorial Policy Checklist.

Statistics

For all statistical analyses, confirm that the following items are present in the figure legend, table legend, main text, or Methods section.

n/a	Confirmed	
☒	☐	The exact sample size (*n*) for each experimental group/condition, given as a discrete number and unit of measurement
☒	☐	A statement on whether measurements were taken from distinct samples or whether the same sample was measured repeatedly
☒	☐	The statistical test(s) used AND whether they are one- or two-sided<br>*Only common tests should be described solely by name; describe more complex techniques in the Methods section.*
☒	☐	A description of all covariates tested
☒	☐	A description of any assumptions or corrections, such as tests of normality and adjustment for multiple comparisons
☒	☐	A full description of the statistical parameters including central tendency (e.g. means) or other basic estimates (e.g. regression coefficient) AND variation (e.g. standard deviation) or associated estimates of uncertainty (e.g. confidence intervals)
☒	☐	For null hypothesis testing, the test statistic (e.g. *F*, *t*, *r*) with confidence intervals, effect sizes, degrees of freedom and *P* value noted<br>*Give P values as exact values whenever suitable.*
☒	☐	For Bayesian analysis, information on the choice of priors and Markov chain Monte Carlo settings
☒	☐	For hierarchical and complex designs, identification of the appropriate level for tests and full reporting of outcomes
☒	☐	Estimates of effect sizes (e.g. Cohen's *d*, Pearson's *r*), indicating how they were calculated

Our web collection on statistics for biologists contains articles on many of the points above.

Software and code

Policy information about availability of computer code

Data collection	RELION v3, CRYOSPARC v3, EPU v2, CTFFIND v4, Gctf v1.06, Motioncor v2
Data analysis	CCP4 v7, COOT v9, RELION v3, CRYOSPARC v3, (only major release numbers given for some programs as minor release numbers changed by automatic updates during work) Xcalibur v4.2, MaxQuant version 1.6.3.4

For manuscripts utilizing custom algorithms or software that are central to the research but not yet described in published literature, software must be made available to editors and reviewers. We strongly encourage code deposition in a community repository (e.g. GitHub). See the Nature Research guidelines for submitting code & software for further information.

Data

Policy information about availability of data

All manuscripts must include a data availability statement. This statement should provide the following information, where applicable:

- Accession codes, unique identifiers, or web links for publicly available datasets
- A list of figures that have associated raw data
- A description of any restrictions on data availability

EM maps and models are deposited in the EMDB and wwPDB under accession codes EMD-12338 and PDB 7NHR (C1 WzcK540M); EMD-12339 and PDB 7NHS (C8 WzcK540M); EMD-12340 (WzcK540M periplasmic localized map); EMD-12360 and PDB 7NII (C1 WzcK540M ADP complex); EMD-12359 and PDB 7NIH (C8 WzcK540M ADP complex); EMD-12353 and PDB 7NIB (C1 WzcK540M4YE); EMD-12349 and PDB 7NI2 (C8 WzcK540M4YE). The mass spectrometry proteomics data have been deposited to the ProteomeXchange Consortium via the PRIDE partner repository with the dataset identifier PXD025820. All constructs are available from the authors until their deposition and release by ADDGENE. The underlying data and original gels are provided in the Extended Data.

Field-specific reporting

Please select the one below that is the best fit for your research. If you are not sure, read the appropriate sections before making your selection.

- [x] Life sciences
- [ ] Behavioural & social sciences
- [ ] Ecological, evolutionary & environmental sciences

For a reference copy of the document with all sections, see nature.com/documents/nr-reporting-summary-flat.pdf

Life sciences study design

All studies must disclose on these points even when the disclosure is negative.

Sample size	not applicable
Data exclusions	No data were excluded
Replication	Structure determination uses thousands of particles. Mass spec also uses many thousands of particles. Immunoblotting experiments comparing the CPS phenotypes of wzc mutations to wild type, were all performed in biological triplicates. These profiles for each analyzed strain or transformant were consistent across replicates. Representative immunoblots were assembled for publication.
Randomization	not applicable
Blinding	not applicable

Reporting for specific materials, systems and methods

We require information from authors about some types of materials, experimental systems and methods used in many studies. Here, indicate whether each material, system or method listed is relevant to your study. If you are not sure if a list item applies to your research, read the appropriate section before selecting a response.

Materials & experimental systems

n/a	Involved in the study	
[ ]	[x]	Antibodies
[ ]	[ ]	Eukaryotic cell lines
[ ]	[ ]	Palaeontology and archaeology
[ ]	[ ]	Animals and other organisms
[ ]	[ ]	Human research participants
[ ]	[ ]	Clinical data
[ ]	[ ]	Dual use research of concern

Methods

n/a	Involved in the study	
[x]	[ ]	ChIP-seq
[x]	[ ]	Flow cytometry
[x]	[ ]	MRI-based neuroimaging

Antibodies

Antibodies used	These are all commercial and well established.
Validation	Antibodies<br>1. Monoclonal anti-polyHistidine-peroxidase antibody produced in mouse was purchased from Sigma (Cat. No. is A7058, https://www.sigmaaldrich.com/catalog/product/sigma/a7058?lang=en&region=GB).<br>2. Monoclonal anti-phosphotyrosine antibody produced in mouse was purchased from Sigma (Cat. No. is P4110, https://www.sigmaaldrich.com/catalog/product/sigma/p4110?lang=en&region=GB&cm_sp=Insite-_-caSrpResults_srpRecs_srpModel_p4110-_-srpRecs3-1).<br>3. HRP conjugated anti-mouse IgG (H+L) antibody was purchased from Promega and used as the secondary antibody for detecting phosphotyrosine (Cat. No. is W402B, https://www.promega.co.uk/products/protein-detection/primary-and-secondary-antibodies/anti_mouse-igg-h-and-l-hrp-conjugate/?catNum=W4021)<br>4. Alkaline phosphatase-conjugated goat anti-rabbit IgG was purchased from Cedarlane (Cat. No. is CLCC43008,https://www.cedarlanelabs.com/products/search?text=CLCC43008&lob=AllProducts&supplierTag=)<br>5. Peroxidase-conjugated goat anti-mouse IgG was purchased from Cedarlane (Cat. No. is 115-036-003, https://www.cedarlanelabs.com/products/search?text=115-036-003&lob=AllProducts&supplierTag=)<br>6. Penta-his antibody was purchased from Qiagen (Cat. No. is 34660, https://www.qiagen.com/ca/search/products?query=34660)

Eukaryotic cell lines

Policy information about cell lines

Cell line source(s)	bacterial cells only
Authentication	not applicable
Mycoplasma contamination	not applicable
Commonly misidentified lines (See ICLAC register)	not applicable

Palaeontology and Archaeology

Specimen provenance	not applicable
Specimen deposition	not applicable
Dating methods	not applicable

☐ Tick this box to confirm that the raw and calibrated dates are available in the paper or in Supplementary Information.

Ethics oversight	not applicable

Note that full information on the approval of the study protocol must also be provided in the manuscript.

Animals and other organisms

Policy information about studies involving animals; ARRIVE guidelines recommended for reporting animal research

Laboratory animals	None
Wild animals	None
Field-collected samples	None
Ethics oversight	not applicable

Note that full information on the approval of the study protocol must also be provided in the manuscript.

Human research participants

Policy information about studies involving human research participants

Population characteristics	None
Recruitment	None
Ethics oversight	not applicable

Note that full information on the approval of the study protocol must also be provided in the manuscript.

Clinical data

Policy information about clinical studies

All manuscripts should comply with the ICMJEguidelines for publication of clinical research and a completedCONSORT checklist must be included with all submissions.

Clinical trial registration	not applicable
Study protocol	not applicable
Data collection	not applicable
Outcomes	not applicable

Dual use research of concern

Policy information about dual use research of concern

Hazards

Could the accidental, deliberate or reckless misuse of agents or technologies generated in the work, or the application of information presented in the manuscript, pose a threat to:

No	Yes	
☒	☐	Public health
☒	☐	National security
☒	☐	Crops and/or livestock
☒	☐	Ecosystems
☒	☐	Any other significant area

Experiments of concern

Does the work involve any of these experiments of concern:

No	Yes	
☒	☐	Demonstrate how to render a vaccine ineffective
☒	☐	Confer resistance to therapeutically useful antibiotics or antiviral agents
☒	☐	Enhance the virulence of a pathogen or render a nonpathogen virulent
☒	☐	Increase transmissibility of a pathogen
☒	☐	Alter the host range of a pathogen
☒	☐	Enable evasion of diagnostic/detection modalities
☒	☐	Enable the weaponization of a biological agent or toxin
☒	☐	Any other potentially harmful combination of experiments and agents
